# Integrative Multi-Omics Analysis and Experiments Validation Identify COX5B as a Novel Therapeutic Target for Lung Adenocarcinoma

**DOI:** 10.32604/or.2025.069889

**Published:** 2025-12-30

**Authors:** Lv Ling, Minying Lu, Ling Ye, Yuanhang Chen, Sheng Lin, Jun Yang, Yu Rong, Guixiong Wu

**Affiliations:** 1 Department of Thoracic Surgery, Panyu Hospital of Traditional Chinese Medicine, Guangzhou, 511400, China; 2 Guangzhou Institute of Cancer Research, The Affiliated Cancer Hospital, Guangzhou Medical University, Guangzhou, 510095, China; 3 Department of Radiation Oncology, Sun Yat-sen Memorial Hospital, Sun Yat-sen University, Guangzhou, 510120, China; 4 Department of Respiratory Medicine, The People’s Hospital of Wuzhou, Wuzhou, 543000, China

**Keywords:** Lung adenocarcinoma (LUAD), cytochrome C oxidase 5B (COX5B), prognosis, proliferation, berberine

## Abstract

**Background:**

A significant proportion of patients still cannot benefit from existing targeted therapies and immunotherapies, making the search for new treatment strategies extremely urgent. In this study, we combined integrate public data analysis with experimental validation to identify novel prognostic biomarkers and therapeutic targets for lung adenocarcinoma (LUAD).

**Methods:**

We analyzed RNA and protein databases to assess the expression levels of cytochrome C oxidase 5B (COX5B) in LUAD. Several computational algorithms were employed to investigate the relationship between COX5B and immune infiltration in LUAD. To further elucidate the role of COX5B in LUAD, we utilized multiple experimental approaches, including quantitative reverse transcription PCR assays, western blot, immunohistochemistry, electron microscopy, flow cytometry, and EdU proliferation assays.

**Results:**

We revealed that COX5B was significantly elevated in LUAD and positively correlated with poor prognosis of LUAD patients. Analysis of co-expression network indicated that COX5B may take part in the intracellular adenosine triphosphate (ATP) synthesis through the oxidative phosphorylation pathway. There was a negative correlation between COX5B expression and immune infiltration in LUAD. Furthermore, we validated that COX5B levels were significantly elevated in both LUAD tissues and cell lines. Specifically, immunohistochemistry (IHC) assays revealed a 2.32-fold increase of COX5B in tumor tissues compared to that in adjacent normal tissues (*p* = 0.0044). Additionally, COX5B knockdown disrupted the redox homeostasis, ultimately suppressed the proliferation of LUAD cells. Subsequent investigations demonstrated that berberine effectively targeted COX5B, diminishing its protein expression and consequently inhibiting cell proliferation and tumor growth in LUAD.

**Conclusions:**

This study established that upregulated COX5B was positive associated with poor patient prognosis in LUAD, elucidating the mechanisms by which berberine targets COX5B to inhibit tumor growth, thereby providing a novel therapeutic target and strategy for the clinical management of LUAD.

## 1 Introduction

Lung cancer is the malignant tumor with the highest incidence and mortality worldwide [1–3]. Approximately 40% of lung cancer cases are classified as lung adenocarcinoma (LUAD) [4]. Despite significant advancements in LUAD clinical management, the 5-year survival rate remains dismal at approximately 15%, primarily due to late-stage diagnosis, easier distant metastasis and therapy resistance [5]. Therefore, identifying new mechanisms of pathogenesis and developing diagnostic and therapeutic targets for LUAD requires immediate attention. For instance, researchers have established a comprehensive bioinformatic and experimental platform, found 4 kinds of biomarkers for cadmium (Cd)-induced LUAD, and revealed the therapeutic efficacy of the natural polyphenol oxyresveratrol (O-RES) against LUAD [6].

Cytochrome C oxidases (COXs), the fourth complex in the electron transport chain, transfers electrons from cytochrome C to oxygen. This reaction leads to water formation couple with pumping protons (H^+^) into the mitochondria intermembrane space [6]. COX is essential for oxidative phosphorylation, as it establishes the electrochemical gradient for ATP production, thereby supplying energy to the cell. The core catalytic subunits of COX, such as mitochondrial cytochrome C oxidase subunit 1 (MT-CO1), MT-CO2, and MT-CO3, are encoded by mitochondrial DNA and synthesized within the mitochondria, where they mediate electron transfer and proton pumping [7]. In contrast, the additional subunits (COX4, COX5A, COX5B, COX6A, COX6B, COX6C, COX7A, COX7B, COX7C, COX8, and NDUEA4/COXEA4) are encoded by nuclear DNA and are involved in regulating the enzyme’s activity and maintaining the stability of the core subunits [8].

Dysregulation of COXs functioned in a range of diseases, including epilepsy and neurodegenerative disorders [9,10]. Increasing research demonstrated that COXs take part in the initiation and progression of tumors [11,12]. For instance, COX4 is overexpressed in thyroid cancer, where its suppression attenuates the p70S6K/pS6 and p-ERK signaling pathways, decreases oxygen consumption and ATP production, and inhibits tumor growth [13]. In breast cancer, SUMOylation of the fusion protein Synaptojanin 2 binding protein-Cytochrome-c oxidase 16 (SYNJ2BP-COX16) promotes tumor progression via phosphorylating DRP1 and increasing mitochondrial fission [14]. COX6B2 facilitates metastasis of pancreatic ductal adenocarcinoma (PDAC) cells by elevating the oxidative phosphorylation levels and activating the purinergic receptor pathway. Fortunately, Metformin was shown to degrade the mRNA level of COX6B2 in order to inhibit the metastatic of PDAC [15].

As a member of COXs, COX5B has been identified as a prognostic indicator in breast cancer [16], clear cell renal cell carcinoma [17], colorectal cancer [18], glioma [19], and so on. However, the comprehensive functions and mechanisms of COX5B need further investigation.

Berberine is an alkaloid derived from the Chinese traditional medicinal herb *Coptis chinensis*. It is used clinically as an antibacterial and antidiarrheal agent [20]. Growing studies noted that berberine induces the apoptosis of tumor cells, and the precise molecular mechanism needs to be discovered. Based on analysis of RNA and protein databases together with experiments validation, and clinical tissue specimens, we aimed to discover the function and its associated molecular mechanisms of COX5B in LUAD. These findings demonstrate that berberine targets COX5B to suppress cell proliferation and tumor growth in LUAD, thereby offering a new potential target and a foundational basis for clinical treatment of this disease.

## 2 Materials and Methods

### 2.1 Data Collection

We downloaded the raw counts data from The Cancer Genome Atlas (TCGA) database (https://cancergenome.nih.gov) and four datasets (GSE40791, GSE10072, GSE27262, and GSE74706) from the Gene Expression Omnibus (GEO) database (https://www.ncbi.nlm.nih.gov/gds, accessed on 25 September 2025). The normalization assay was performed using the DESeqDataSetFromMatrix function from the ‘DESeq2_1.39.8’ R_4.2.1 package with the variance stabilizing transformation (VST) method. Moreover, the transcriptomic and clinical data of LUAD patients were obtained from the TCGA database. Then, the protein levels of COX5B in LUAD were analyzed by The Human Protein Atlas (https://www.proteinatlas.org) and UALCAN (https://ualcan.path.uab.edu), which provide pre-normalized and pre-corrected data. The expression patterns of COX5B in pan-cancers were analyzed from TCGA and UALCAN.

### 2.2 The Analysis of the COX5B Expression Pattern

Briefly, we evaluated the expression levels of COX5B in LUAD tissues and normal lung tissues from TCGA and GEO databases using the Mann-Whitney U test. The UALCAN database was employed to assess the protein expression pattern of COX5B in LUAD. Furthermore, the results of Immunohistochemistry (IHC) assays for COX5B in LUAD and normal lung tissue were obtained through the Human Protein Atlas.

### 2.3 Exploring the Clinical Significance of Elevated COX5B in LUAD

Based on the average expression level of COX5B, the LUAD patients from the TCGA database were divided into high and low groups. Chi-square test was applied to estimate the relationship between COX5B expression and the clinical parameters of LUAD. Subsequently, the correlation of COX5B expression and the prognosis of patients with LUAD was determined via Kaplan-Meier analysis. Both univariate and multivariate Cox regression analyses were conducted to evaluate the impact of COX5B expression and other clinical parameters on outcomes of patients. A nomogram was developed utilizing the ‘RMS_6.7-0’ and ‘survival_3.5-7’ packages, followed by calibration to assess prediction accuracy. Additionally, Receiver Operating Characteristic (ROC) curves were generated via the ‘timeROC’ package to detect the diagnostic value of COX5B in the prognosis of LUAD patients.

### 2.4 Functional Enrichment Analysis

The LinkedOmics database (http://www.linkedomics.org/login.php, accessed on 25 September 2025) serves as a comprehensive analytical resource. We performed the co-expression analysis of COX5B in the TCGA-LUAD dataset via the Spearman correlation coefficient. Gene Ontology (GO) and Kyoto Encyclopedia of Genes and Genomes (KEGG) pathway analysis were carried out with FDR < 0.05.

The COX5B-protein interaction network was implemented on the STRING database (https://cn.string-db.org/).

### 2.5 Discovering the Effect of COX5B on Immune Infiltration

The infiltration of 24 different types of immune cells in LUAD was analyzed using single-sample gene set enrichment analysis (ssGSEA) assays through the ‘GSVA_1.48.3’ package. Meanwhile, the ImmuneScore, ESTIMATEScore, and StromalScore approaches were utilized to explore the correlation between COX5B and immune cell infiltration by Spearman correlation analysis, with the findings visualized via the ‘ggplot2 _3.4.4’ package. In addition, the Cibersort algorithm was employed to analyze the correlation between COX5B and immune invasion in LUAD.

### 2.6 Identification of a Potential Therapeutic Target of COX5B

Potential COX5B binding agents were identified from the open-source tool DGIdb (https://dgidb.org/). The structural information for these compounds was sourced from the PubChem database (https://pubchem.ncbi.nlm.nih.gov/), while the COX5B protein structure was retrieved from the PDB database (www.rcsb.org/). Molecular docking of the receptor protein with the ligand small molecules was conducted using AutoDock Vina 1.1.2. The function used is: Score = ∑[Weight_term ∗ Interaction_term]. The related parameters were listed as below: num_modes = 20, energy_range = 3, exhaustiveness = 32. and the docking outcomes were analyzed using PLIP, with visualizations created in PyMOL v2.5.0.

### 2.7 Cell and Cell Culture

Human normal lung epithelial cells BEAS-2B and LUAD cell lines (A549, H1299, H1975, and HCC827) were acquired from the American Type Culture Collection (ATCC, Manassas, VA, USA). All cells were cultured in 1640 medium (Gibco, Grand Island, NY, USA) supplemented with 10% Fetal Bovine Serum (Gibco, Grand Island, USA). According to the standard protocols, these cells were cultured in an incubator at 37°C with 5% CO_2_. All cells were authenticated by short tandem repeat (STR) analysis. All cells were free of mycoplasma contamination.

### 2.8 Tissue Samples

A cohort of 24 cases of LUAD tissues and paired normal tissues, another cohort of 13 cases of normal tissues and 64 cases of LUAD tissues, were collected from the Affiliated Cancer Hospital of Guangzhou Medical University with written informed consent (ethics approval number: 2021-SZ21).

### 2.9 Transfection, RNA Extraction and Quantitative Reverse Transcription Polymerase Chain Reaction (qRT-PCR) Assays

The plasmids of COX5B overexpression, three shRNAs targeting COX5B and control were obtained from Gene Biological Co., Ltd. (Shanghai, China). The shRNA control plasmid included a scramble sequence (5^′^-TTCTCCGAACGTGTCACGT-3^′^). The plasmid was transfected into H1299 and H1975 cells with Lipofectamine™ 3000 (Invitrogen, Carlsbad, CA, USA) following the manufacturer’s instructions. Briefly, 4 h after transfection, cells were recovered in 1640 medium with 10% FBS. 24 h later, 1 μg/mL of a eukaryotic selection antibiotic was added to establish a stably modified cell line. Approximately one month later, the total RNAs were extracted using the RNA extraction kit (#R6834, Omega Bio-Tek, Norcross, GA, USA), and then reverse transcribed into cDNA with the RevertAid First Strand cDNA Synthesis Kit (#K1622, Thermo Fisher, Waltham, MA, USA). qRT-PCR assays were performed with PowerUp SYBR Green Master Mix (Thermo Fisher). GAPDH was used as an internal control. The sequences of genes are listed below: GAPDH-F: 5^′^-GTCTCCTCTGACTTCAACAGCG-3^′^, GAPDH-R: 5^′^-ACCACCCTGTTGCTGTAGCCAA-3^′^; COX5B-F: 5^′^-GGAGATCATGCTGGCTGCAAAG-3^′^, COX5B-R: 5^′^-GCAGCCTACTATTCTCTTGTTGG-3^′^. The relative levels of gene expression were calculated by the 2^−ΔΔCt^ method. All assays were conducted at least three times.

### 2.10 Western Blot Assays

The proteins from H1299 and H1975 cells were obtained with RIPA lysis buffer (#89901, Thermo Fisher, Waltham, MA, USA) and protease inhibitors (#78430, Thermo Fisher, Waltham, MA, USA). The concentration of these proteins was measured via the BCA kit (PI23225, Thermo Fisher, Waltham, MA, USA). A total of 30 μg protein was loaded per well. After SDS-PAGE electrophoresis, the proteins were transferred to a PVDF membrane, followed by 5% Bovine Serum Albumin (BSA, PP2100-100, Thermo Fisher, Waltham, MA, USA) for 2 h, specific primary antibodies overnight, and a secondary antibody for another 2 h. Finally, protein bands were detected via an ECL chemiluminescence kit (#32106, ThermoFisher, Waltham, MA, USA). The ECL luminescence signals are acquired by a Tanon imaging system (Tanon 5200Multi, Tanon, Shanghai, China).

The BSA reagent, HRP-conjugated secondary antibody (1:5000, SA00001), GAPDH antibody (1:1000, 60004-1-Ig), and COX5B antibody (1:1000, 11418-2-AP) were all acquired from Proteintech Co., Ltd. (Wuhan, China). All assays were conducted at least three times.

### 2.11 EdU Proliferation Assays

This study was performed by BeyoClick™ EdU Cell Proliferation Kit with AF555 (C0075S, Beyotime, Shanghai, China). H1299 and H1975 cells were seeded into a 24-well plate at a density of 40,000 cells per well. Once the cell confluence reached 80%, the EdU working solution was added. 2 h later, 1 mL of 4% paraformaldehyde, 1 mL of 0.3% Triton X-100 permeabilization solution, and 0.5 mL of Click reaction solution were sequentially added. Each incubation lasted for 30 min. At last, the nucleus was stained with Hoechst 33,342. A fluorescence microscope Axio Observer (ZEISS, Oberkochen, Germany) was utilized to take photos.

### 2.12 Colony Formation Assays

H1299 and H1975 cells were plated into six-well plates at a density of 500 cells per well. The fresh culture medium was added every 2 or 3 days. Two weeks later, the cells were fixed with pre-cooled absolute methanol and stained with 1% crystal violet. Subsequently, the cells were photographed and counted.

### 2.13 Cell Viability Assays

A total of 1000 H1299 and H1975 cells per well were seeded into a 96-well plate. At 0, 1, 2, 3, 4, 5 days, 20 μL of MTS-[3-(4,5-dimethylthiazol-2-yl)-5-(3-carboxymethoxyphenyl)-2-(4-sulfophenyl)-2H-tetrazolium] (Promega, Madison, WI, USA) was added to each well. These cells were incubated at 37°C for 3 h. The absorbance at 490 nm was measured by a multifunctional microplate reader BioTek Synergy 2 (Agilent Technologies, Santa Clara, CA, USA).

In another experiment, H1299 and H1975 cells were plated at 3000 cells per well in a 96-well plate. Berberine (S9046) was obtained from Selleck (Houston, TX, USA). Varying concentrations of berberine (0, 30, 60, 90, 120, and 150 μM) were used with cells for 72 h. Then, 20 μL of MTS was added to each well and incubated for 3 h at 37°C. The absorbance at 490 nm was measured to evaluate cellular activity in each group. The IC50 values of these cells to berberine was calculated by GraphPad Prism 10.1.2.

For discover the effect of berberine on COX5B, H1299 and H1975 cells were exposed to 0, 10, 20, and 30 μM berberine for 48 h. Then these cells were utilized to detect the mRNA and protein levels of COX5B.

### 2.14 ROS Measurement

The ROS detection kit (S0033S) was provided by Beyotime Co., Ltd. (Shanghai, China). H1299 and H1975 cells were seeded in a 6-well plate at a density of 300,000 cells per well. The next day, the cells were incubated with DCFH-DA detection buffer in serum-free culture medium for 20 min. After washing, the cells were collected and analyzed for ROS levels using flow cytometry BD FACSCanto II (BD biosciences, San Jose, CA, USA).

### 2.15 Detection of ATP Levels

The ATP levels of each group were detected by the Enhanced ATP Assay Kit (S0027, Beyotime, Shanghai, China). A total of 300,000 H1975 and H1299 cells were seeded in a 6-well plate per well, and once they reached approximately 80% confluence, they were lysed. The resulting supernatant was centrifuged at 12,000× *g* for 5 min at 4°C using a centrifuge from Eppendorf (Hamburg, Germany) to remove cells and debris.

For further analysis. Subsequently, 100 μL of the ATP assay working solution was added to each well of the detection plate, followed by the addition of 20 μL test sample. The chemiluminescence level of each well was measured by a multifunctional microplate reader.

### 2.16 Electron Microscopy Assays

H1299 and H1975 cells were harvested and treated with 2.5% glutaraldehyde fixative. After that, the samples were sent to the Shiyanjia Lab (www.shiyanjia.com, Chengdu, China) to take a photo of mitochondrial morphology by electron microscopy.

### 2.17 Immunohistochemistry (IHC) Assays

The IHC kit (KIT-5001) was supplied by Maixin Biological Co., Ltd. (Fuzhou, China). The samples were paraffin-embedded, dewaxed and boiled for antigen retrieval in citric acid buffer. Subsequently, these slides were soaked in 3% H_2_O_2_ and blocked with 10% normal goat serum. Then, the slides were incubated with primary antibody against COX5B (Proteintech, 11418-2-AP, 1:500) overnight at 4°C, and followed by secondary antibody. At last, these slides were stained with diaminobenzidine (DAB) reagent and counterstained with hematoxylin. Images were captured with Panoramic 250 FLASH (3DHistech, Budapest, Hungary) and assessed with Case Viewer software 2.4.1 (3DHistech, Budapest, Hungary). All IHC staining was evaluated and scored by at least two independent pathologists. The scores of each section were multiplied to give a final score of 0–12, and the tumors were finally determined as negative (−), score 0; lower expression (+), score ≤ 4; moderate expression (++), score 5–8; and high expression (+++), score 9–12.

### 2.18 Animal Experiments

This study was approved by the Animal Ethics Committee of the People’s Hospital of Wuzhou (ethics approval number: 2023033). All procedures were conducted in accordance with the Animal Ethics Committee’s guidelines.10 BALB/C nude mice (4 weeks, female, 18–22 g) were obtained from the Guangdong Medical Animal Center (Foshan, China). These mice were maintained under specific pathogen-free (SPF) conditions. A total of 2 × 10^5^ cells was subcutaneously injected into each nude mouse. Tumor size was assessed every three days, and the tumor volume was calculated with the formula V = 1/2 LW^2^. After 3 days, the tumor volume reached approximately 50 mm^3^, the nude mice were randomly divided into two groups (*n* = 5). Intraperitoneal injections of 1 × PBS (pH = 7.2) or berberine (50 mg/kg) were given every two days, for just 4 cycles. Institutional Animal Care and Use Committee (IACUC) stipulated that the tumor volume couldn’t exceed 2000 mm^3^, and none of the mice exceeded these limits. After 18 days, we ended the experiment. These 10 mice were sacrificed with carbon dioxide. The tumor size and weight were measured to evaluate the effect of berberine. The Ki67 and COX5B expression of each group was measured via IHC assays.

### 2.19 Statistical Analysis

All the Data were represented as mean ± standard deviation (M ± SD). Differences between the two groups were assessed using an independent sample *t*-test or a paired sample *t*-test. Chi-square test was applied for comparisons involving multiple groups. *p* < 0.05 was deemed statistically significant.

## 3 Results

### 3.1 Upregulated COX5B Independently Predicts the Poor Prognosis of Patients with LUAD from Several Database Analyses

To discover the expression pattern of COX5B across various cancers, we searched the TCGA database. As illustrated in Fig. S1A, COX5B was highly expressed in several malignancies, including bladder urothelial carcinoma (BLCA), breast invasive carcinoma (BRCA), cervical squamous cell carcinoma and endocervical adenocarcinoma (CESC), cholangiocarcinoma (CHOL), kidney renal papillary cell carcinoma (KIRP), liver hepatocellular carcinoma (LIHC), lung adenocarcinoma (LUAD), mesothelioma (MESO), prostate adenocarcinoma (PRAD), and uterine corpus endometrial carcinoma (UCEC). Oppositely, COX5B exhibited a lower expression in kidney chromophobe (KICH) and kidney renal clear cell carcinoma (KIRC). Furthermore, we estimated the protein expression levels of COX5B via CPTAC database. The results revealed that COX5B was significantly elevated in ovarian cancer, UCEC and lung cancer compared to normal tissues. On the other hand, COX5B expression was decreased in breast cancer, colon cancer, clear cell renal cell carcinoma (RCC), pancreatic adenocarcinoma (PAAD), head and neck cancers, glioblastoma, and liver cancer (Fig. S1B). From TCGA database, Kaplan-Meier analysis noted that COX5B was correlated with overall survival, progression-free interval, and disease-specific survival in several cancers (Fig. S1C–E). These data suggested that COX5B was dysregulated across various malignancies.

**Figure 1:**
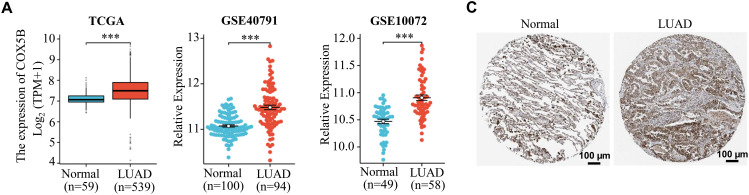

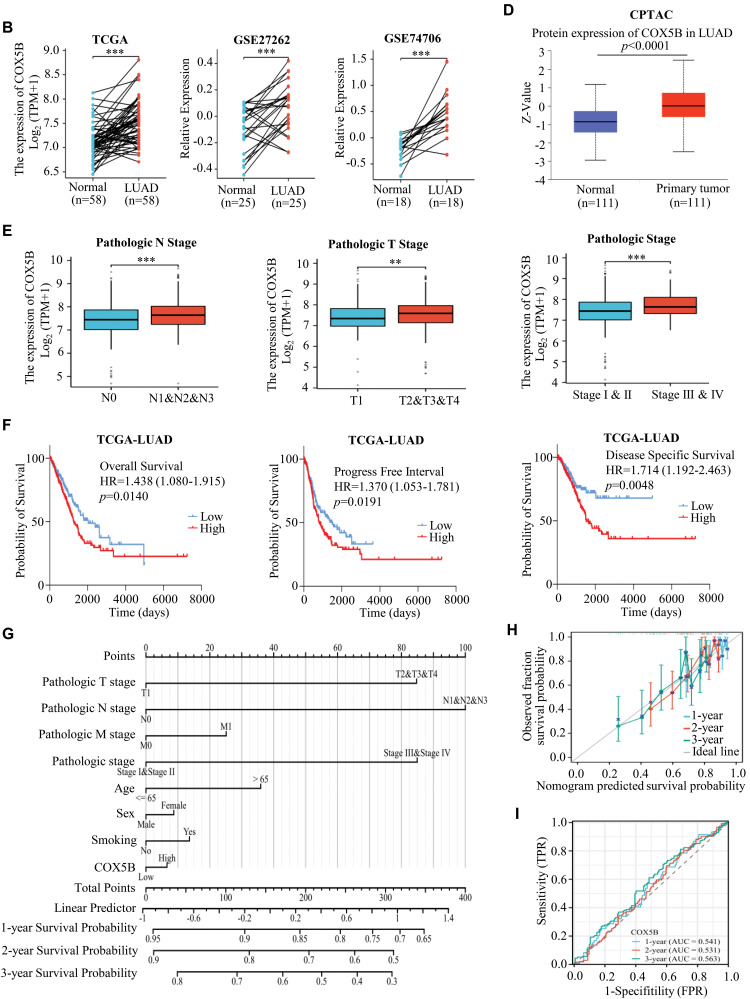
Bioinformatics analysis of the expression pattern and clinical significance of COX5B in LUAD. (**A**) The expression pattern of COX5B in LUAD was observed in the TCGA database and GEO datasets (GSE40791 and GSE10072), vs. Normal, ***p < 0.001. (**B**) TCGA database and GEO datasets (GSE27262 and GSE74706) illustrated that COX5B was highly expressed in LUAD tissues compared with that in paired normal tissues, vs. Normal, ***p < 0.001. The Human Protein Atlas database (**C**) and UALCAN database (**D**) evaluated the protein expression levels of COX5B in LUAD and normal lung tissue. (**E**) Analysis of COX5B expression with different lymph node metastasis, pathological T stage, and pathological stage in TCGA-LUAD cohort, vs. N0, T1, and Stage I & II, **p < 0.01, ***p < 0.001. (**F**) The Kaplan-Meier analysis was carried out to assess the relationship between COX5B expression and overall survival rate, progression-free interval, and disease-specific survival of patients with LUAD, respectively. A nomogram (**G**), calibration curve (**H**), and time-dependent ROC curve (**I**) were established to determine the diagnostic value of COX5B in LUAD

Next, we laid attention to COX5B expression in LUAD. As presented in Fig. 1A, COX5B was significantly overexpressed in 539 LUAD tissues compared to 59 adjacent normal tissues in the TCGA database. GEO datasets (GSE40791 and GSE10072) exhibited a similar result. As confirmed in Fig. 1B, COX5B was markedly higher in LUAD tissues than in paired adjacent tissues from both the TCGA and GEO datasets (GSE27262 and GSE74706). Given the mRNA levels of COX5B in LUAD, we further evaluated the protein levels of COX5B in LUAD. The analysis of Human Protein Atlas database revealed that COX5B levels were considerably higher in LUAD than those in normal lung tissues (Fig. 1C). Consistent results were also supported by the CPTAC protein database (Fig. 1D). From the TCGA-LUAD cohort, we employed the chi-square test to explore the association between COX5B and various clinical characteristics. As shown in Table 1, COX5B expression was significantly related to lymph node metastasis, pathological T stage, and clinicopathological stage. The detailed results were shown in Fig. 1E. Moreover, there was a significant positive correlation between COX5B expression and overall survival, progression-free interval, and disease-specific survival of patients with LUAD (Fig. 1F). As exhibited in Table 2, univariate analysis revealed that COX5B was significantly associated with the overall survival rate of LUAD patients [hazard ratio (HR) = 1. 452, 95% confidence interval (CI) 1.085–1.944, *p* = 0.012]. We generated a nomogram via integrating COX5B with all clinicopathological parameters and yielded a C-index of 0.682 (Fig. 1G). Calibration curves for the 1-year, 2-year and 3-year overall survival probabilities showed good consistency between the nomogram predictions and the results observed in the TCGA database (Fig. 1H). A ROC curve analysis illustrated that the predictive accuracy of COX5B for 1-year, 2-year and 3-year survival was 0.541, 0.531, and 0.563, respectively (Fig. 1I). In summary, these results indicate that COX5B is highly expressed in LUAD, is closely associated with lymph node metastasis, T stage, and clinicopathological stage, and serves as an independent prognostic marker for LUAD.

**Table 1:** Analysis of the expression of COX5B and the clinical parameters in LUAD from the TCGA database

Characteristics	Low expression		High expression		(df, *n*) = *χ*^2^	*p* value
	*n*	(%)	*n*	(%)		
**Pathologic T stage**					(1536) = 9.022	0.003
T1	104	19.40	72	13.43		
T2 & T3 & T4	163	30.41	197	36.75		
**Pathologic N stage**					(1523) = 9.618	0.002
N0	188	35.95	162	30.98		
N1 & N2 & N3	68	13.00	105	20.08		
**Pathologic M stage**					(1390) = 0.514	0.473
M0	173	44.36	192	49.23		
M1	10	2.56	15	3.85		
**Pathologic stage**					(1511) = 7.534	0.006
Stage I & II	205	40.12	196	38.36		
Stage III & IV	40	7.83	70	13.70		
**Sex**					(1539) = 0.534	0.465
Female	140	25.97	149	27.64		
Male	129	23.93	121	22.45		
**Age**					(1520) = 0.497	0.481
>65	129	24.81	134	25.77		
≤65	134	25.77	123	23.65		
**Smoking**					(1525) = 0.011	0.916
No	39	7.43	38	7.23		
Yes	224	42.67	224	42.67		

Note: COX5B, cytochrome C oxidase 5B; LUAD, lung adenocarcinoma; TCGA, The Cancer Genome Atlas; df, degree of freedom.

**Table 2:** Univariate and multivariable results of overall survival identifying the prognostic value of COX5B

Characteristics	Total (N)	Univariate analysis		Multivariate analysis	
		Hazard ratio (95% CI)	*p* value	Hazard ratio (95% CI)	*p* value
**Pathologic T stage**	504				
T1	169	Reference		Reference	
T2 & T3 & T4	335	1.665 (1.182–2.345)	0.004	1.589 (1.012–2.493)	0.044
**Pathologic N stage**	495				
N0	327	Reference		Reference	
N1 & N2 &N3	168	2.582 (1.922–3.470)	<0.001	1.963 (1.328–2.900)	<0.001
**Pathologic M stage**	363				
M0	338	Reference		Reference	
M1	25	2.143 (1.251–3.672)	0.006	1.133 (0.597–2.150)	0.703
**Pathologic stage**	499				
Stage I & II	392	Reference		Reference	
Stage III & IV	107	2.662 (1.953–3.628)	<0.001	1.785 (1.152–2.766)	0.010
**Sex**	507				
Female	272	Reference			
Male	235	1.070 (0.800–1.431)	0.648		
**Age**	497				
≤65	239	Reference			
>65	258	1.213 (0.904–1.629)	0.198		
**Smoking**	493				
No	72	Reference			
Yes	421	0.914 (0.605–1.379)	0.668		
**COX5B**	507				
Low	255	Reference		Reference	
High	252	1.416 (1.054–1.902)	0.021	1.155 (0.815–1.638)	0.417

Note: COX5B, cytochrome C oxidase 5B; CI, confidence interval.

### 3.2 Analysis of COX5B Co-Expression Network in LUAD

**Figure 2:**
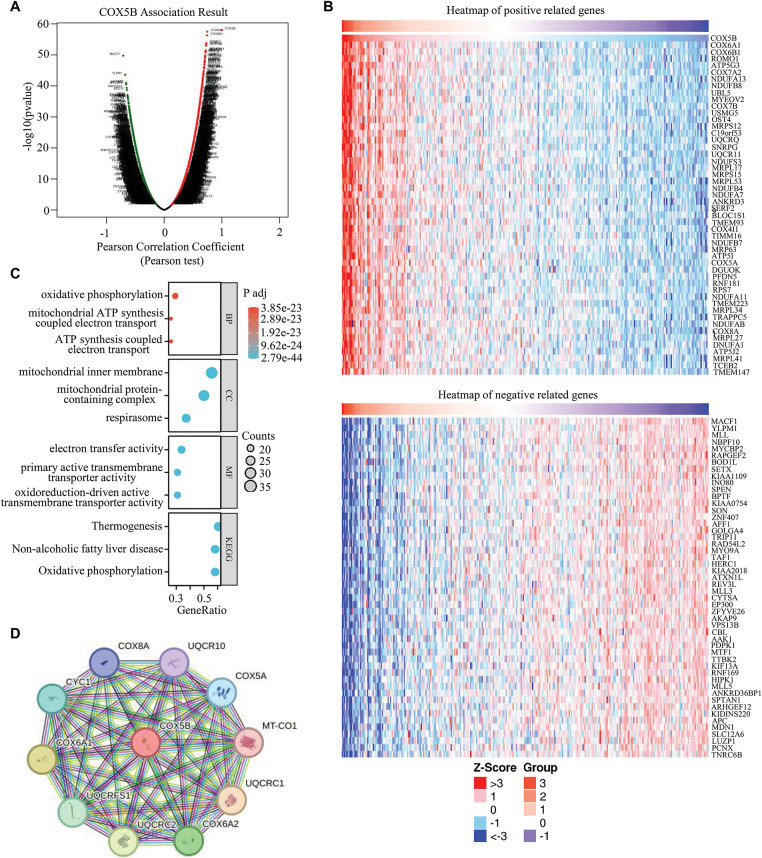
Analysis of the co-expression network for COX5B. (**A**) A volcano map was drawn to show the 19,962 genes co-expressed with COX5B in LUAD. (**B**) Heatmap of the top 100 genes that are positively and negatively correlated with COX5B in LUAD. (**C**) A bubble chart was generated to represent the enrichment analysis of genes associated with COX5B expression. (**D**) The STRING database was utilized to identify the proteins interacting with COX5B.

To explore the potential role and mechanism of COX5B in LUAD, we analyzed the co-expression genes of COX5B using the TCGA-LUAD dataset. As shown in Fig. 2A, we identified 19,962 genes exhibiting a significant association with COX5B in LUAD. Subsequently, the heatmaps of top 100 genes having positive and negative correlations with COX5B were presented in Fig. 2B. To uncover the functional role of COX5B, we conducted GO and KEGG pathway analyses with the mentioned 100 related genes. GO results depicted that these related genes were mainly involved in oxidative phosphorylation and mitochondrial ATP synthesis electron transport chain (Fig. 2C). The KEGG analysis revealed that these co-expressed genes were largely associated with thermogenesis, non-alcoholic fatty liver disease, and oxidative phosphorylation (Fig. 2C). At last, the STRING database was utilized to create a protein-protein interaction network for COX5B (Fig. 2D). Collectively, these findings imply that COX5B may play a function role in the oxidative phosphorylation pathway and ATP synthesis.

### 3.3 COX5B Correlates Inversely with Immune Infiltration in LUAD

**Figure 3:**
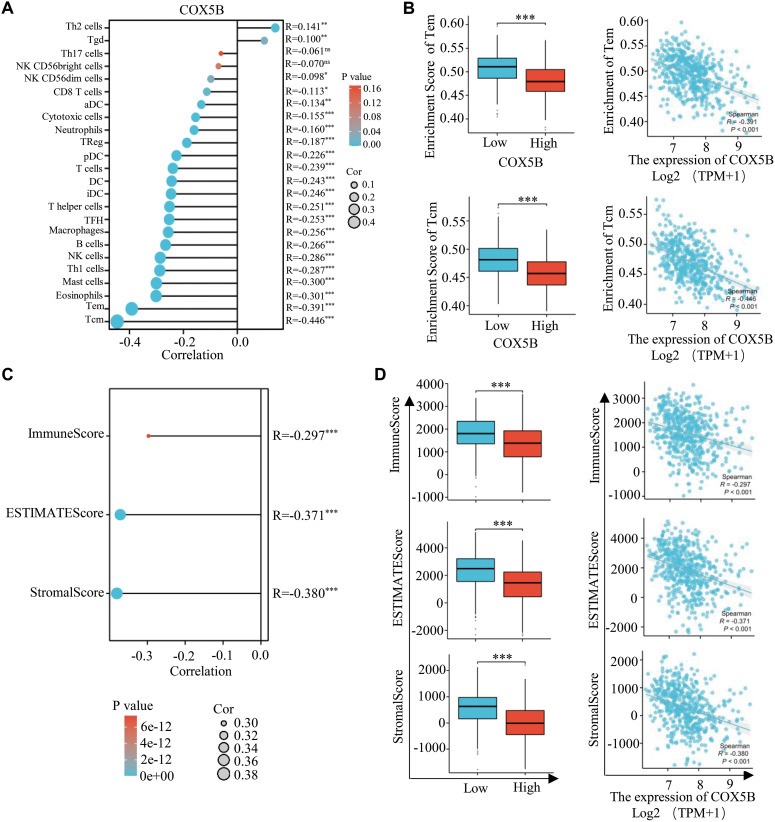

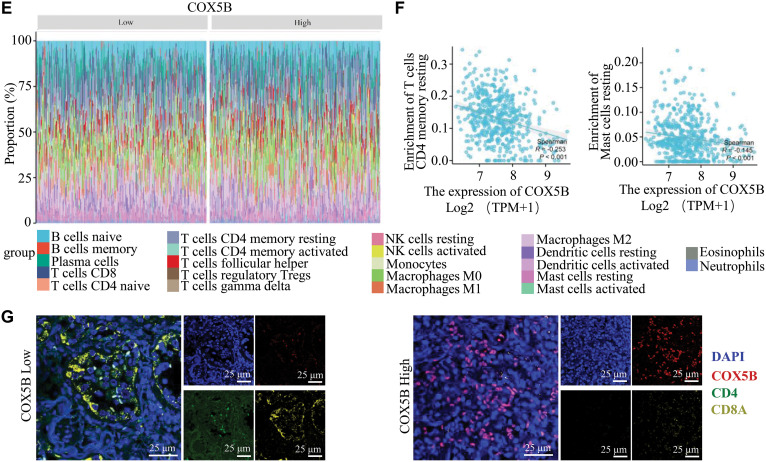
COX5B shows a negative correlation with immune Infiltration in LUAD. (**A**) The relationship between COX5B and immune cell infiltration in LUAD was assessed using the ssGSEA assay, ns, non-significant; *p < 0.05, **p < 0.01, ***p < 0.001. (**B**) The relationship between COX5B expression and infiltration of Tem and Tcm cells in LUAD was analyzed, respectively. ***p < 0.001. (**C**,**D**) The association of COX5B with immune infiltration in LUAD was evaluated by ImmuneScore, ESTIMATEScore, and StromalScore algorithms, ***p < 0.001. (**E**) The Cibersort algorithm was employed to analyze the correlation between COX5B and immune invasion in LUAD. (**F**) An examination of the relationship between COX5B expression and the infiltration of resting CD4^+^ memory T cells and resting mast cells in LUAD was conducted. (**G**) The relationship between COX5B and CD4^+^ T cells, CD8^+^ T cells was estimated in LUAD tissues via multiplex immunohistochemistry (mIHC) assays

Through ssGSEA algorithm, we indicated that COX5B was related to various immune cell infiltrations. Particularly, it was negatively correlated with the infiltration of central memory T cells (Tcm) and effector memory T cells (Tem) (Fig. 3A,B). Following this, the ImmuneScore, ESTIMATEScore, and StromalScore approaches indicated a negative correlation between COX5B and immune invasion in LUAD (Fig. 3C,D). Consistently, the Cibersort algorithm confirmed an inverse effect of COX5B on the infiltration of resting CD4^+^ memory T cells (r = −0.253) and resting mast cells (r = −0.145), respectively (Fig. 3E,F). To verify this, we performed multiplex immunohistochemistry (mIHC) on clinical tissue samples from LUAD patients. The results showed that tissues with high COX5B expression exhibited lower levels of both CD4 and CD8A, whereas the tissues with low COX5B expression did the opposite (Fig. 3G). Additionally, COX5B exhibited a negative correlation with immune cell infiltration in LUAD, LUSC, PAAD, and THCA. While showing an opposite trend in UVM, THYM and LAML (Fig. S1F). Overall, these findings propose that COX5B may show a negative correlation with immune infiltration in LUAD.

### 3.4 Increased COX5B Confers Proliferation of LUAD Cells

**Figure 4:**
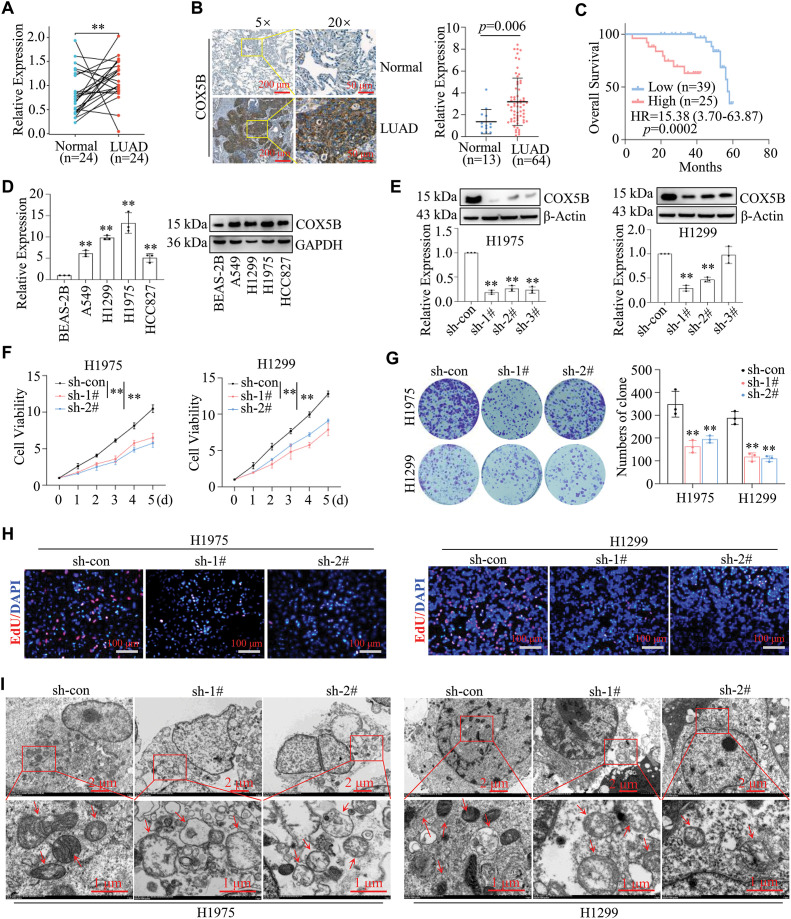

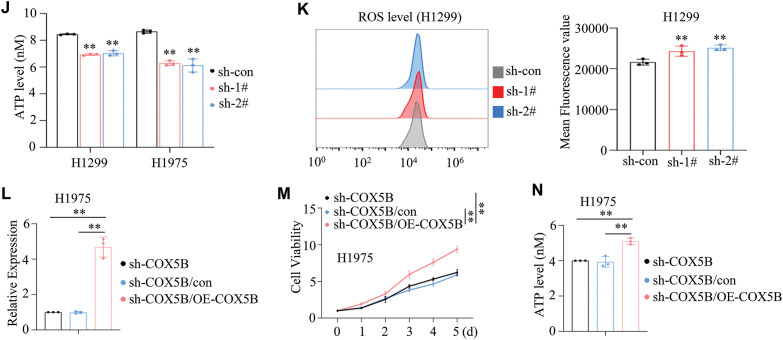
Validation of COX5B expression patterns, roles, and potential mechanisms in LUAD. (**A**) RNAs were extracted from 24 pairs of LUAD tissues and adjacent normal tissues, and qRT-PCR assays were performed to determine the expression levels of COX5B, **p < 0.01. (**B**) The protein levels of COX5B in 13 normal tissues and 64 LUAD tissues were assessed by IHC assays. (**C**) The relationship between COX5B expression and the overall survival of LUAD patients was analyzed via Kaplan-Meier analysis. (**D**) The expression levels of COX5B in normal lung epithelial cells (BEAS-2B) and LUAD cell lines (A549, H1299, H1975, and HCC827) were measured using qRT-PCR and WB assays, vs. BEAS-2B, **p < 0.01. (**E**–**K**) shRNAs targeting COX5B were transfected into H1299 and H1975 cells, respectively. qRT-PCR and WB assays (**E**) were applied to evaluate the expression of COX5B vs. sh-con, **p < 0.01. The cell viability assays (**F**), colony formation assays (**G**), and EdU proliferation assays (**H**) were carried out in the mentioned cells, vs. sh-con, **p < 0.01. (**I**) The mitochondrial morphology of these cells was examined using electron microscopy. The red arrows indicate mitochondria. The ATP levels (**J**) and ROS levels (**K**) were measured in these cells, vs. sh-con, **p < 0.01. (**L–N**) COX5B overexpression plasmid and con plasmid were transfected into H1975 cells with the knockdown of COX5B (sh-1#), respectively. The COX5B expression (**L**), the cell viability (**M**) and the ATP levels (**N**) of the mentioned cells were measured, vs. sh-COX5B, vs. sh-COX5B/con, **p < 0.01

Since we have determined the expression profile, function, and potential mechanisms of COX5B in LUAD from several databases, experimental data are urgently needed to validate these findings. Initially, the results of qRT-PCR assays revealed that COX5B was markedly elevated in 24 LUAD tissues compared with that in paired normal tissues (Fig. 4A). Similarly, IHC assays confirmed that the protein levels of COX5B were significantly higher in 64 LUAD tissues than those in 13 normal tissue samples (Fig. 4B). As shown in Fig. 4C, high expression of COX5B was associated with worse overall survival (HR = 15.38, 95% CI: 3.70–63.87, *p* = 0.0002). Further, we verified that COX5B was notably greater in LUAD cell lines (A549, H1299, H1975, and HCC827) than in the normal lung epithelial cell line BEAS-2B via qRT-PCR and WB assays (Fig. 4D). We focused on H1299 and H1975 cells for their highest expression of COX5B. To identify the role of COX5B in LUAD cells, shRNAs targeting COX5B were transfected into H1975 and H1299 cells. sh-1# and sh-2# were used in the following study for their best inhibition efficiency on COX5B (Fig. 4E). Moreover, we illustrated that COX5B knockdown significantly suppressed the viability of H1299 and H1975 cells (Fig. 4F). Consistent findings were obtained by colony formation assays (Fig. 4G) and EdU proliferation assays (Fig. 4H). Based on the GO and KEGG pathway analysis, we conducted electron microscopy analysis in LUAD cells. Compared to the control group, the mitochondria in COX5B-decreasing cells were swollen, ruptured, and exhibited a loss of cristae (Fig. 4I). The ATP levels in these cells were significantly diminished, too (Fig. 4J), while ROS levels were markedly enhanced (Fig. 4K). To investigate whether the effects of COX5B knockdown were specific, we performed a rescue experiment by restoring COX5B in COX5B-deficient H1975 cells (Fig. 4L). MTS results noted that COX5B re-overexpression significantly rescued the impaired cell viability caused by decreasing COX5B (Fig. 4M). Consistent with this finding, the ATP level, which was reduced upon COX5B knockdown, was also restored upon re-expression of COX5B (Fig. 4N). Taken together, these data strongly suggested that knocking down COX5B may alter mitochondrial structure and ATP production, disrupt the redox balance, thereby inhibiting the proliferation of LUAD cells.

### 3.5 COX5B Is a Potential Therapeutic Target in LUAD

**Figure 5:**
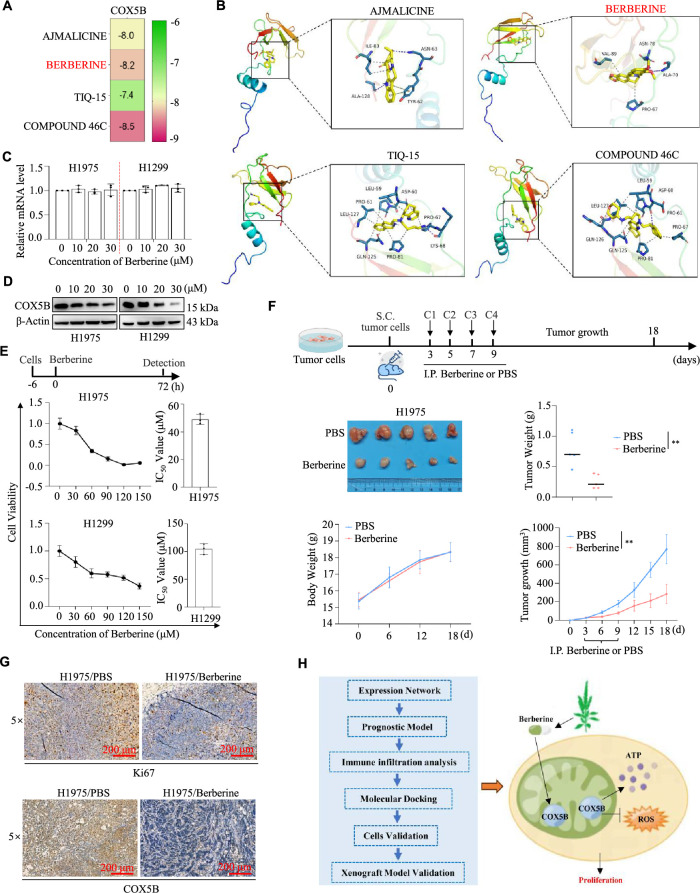
Berberine targeting COX5B suppressed LUAD progression. (**A**) Through the online tool DGIdb (https://dgidb.org/), 4 kinds of compounds (AJMALICINE, BERBERINE, TIQ-15, and COMPOUND 46C) were identified to bind with COX5B. (**B**) The specific binding sites of the four drugs on COX5B were identified and visualized. (**C**,**D**) H1299 and H1975 cells were exposed to 0, 10, 20, and 30 μM berberine for 48 h, and COX5B expression was assessed using qRT-PCR (**C**) and WB assays (**D**). (**E**) H1975 and H1299 cells were treated with 0, 30, 60, 90, 120, and 150 μM berberine for 72 h, followed by MTS assay to evaluate cell proliferation and the calculation of the IC_50_ value. (**F**) H1975 cells were subcutaneously injected into nude mice, and once the tumor volume reached 50 mm^3^, PBS or berberine was administered intraperitoneally every two days to monitor tumor growth. vs. PBS, **p < 0.01. (**G**) IHC assays were performed to measure the expression level of Ki67 and COX5B in the mentioned tumor tissues. (**H**) The diagram of this study was shown.

Since the function and mechanisms of COX5B in LUAD have been explored, we focused on the potential treatment targeting COX5B for LUAD. Utilizing drug library screening and molecular docking techniques, we briefly discovered that there were four drugs (including ajmalicine, berberine, tiq-15, and compound 46c) showed a specific affinity for COX5B, with the docking scores are −8.0, −8.2, −7.4, and −8.5 kcal/mol, respectively (Fig. 5A). As shown in Fig. 5B, ajmalicine formed hydrogen bonds with the active residues ASN-63 and ALA-128 of protein COX5B. Similarly, berberine bound to ASN-78 and ALA-70 of COX5B via hydrogen bonding. Tiq-15 interacted with LYS-68 of COX5B through a hydrogen bond, while compound 46c formed a hydrogen bond with ASP-60 of COX5B. Berberine is a common drug used for intestinal infection and bacillary dysentery [21]. Here, we selected berberine for further study due to its well-established safety and pharmacokinetic profile. This recognized advantage could facilitate its rapid repurposing for LUAD therapy. As expected, berberine sharply decreased the protein but not mRNA levels of COX5B in H1299 and H1975 cells (Fig. 5C,D). Subsequently, different doses of berberine treated H1299 and H1975 cells, and the viability of each group was measured. The results determined an IC_50_ of 104.70 ± 9.45 μM for H1299 cells and 49.09 ± 3.64 μM for H1975 cells (Fig. 5E). To further investigate the influence of berberine on tumor growth *in vivo*, H1975 cells were subcutaneously injected into BALB/c nude mice to establish a subcutaneous mouse model. Consistently, berberine significantly inhibited both tumor growth and weight compared with the PBS group (Fig. 5F). There were no significant differences in body weight between the two groups (Fig. 5F). As shown in Fig. 5G, berberine suppressed COX5B and reduced tumor proliferation via IHC assays. Therefore, these findings indicate that berberine selectively binds to COX5B, downregulates its protein expression, and effectively hampers the proliferation and tumor growth of LUAD.

## 4 Discussion

Mitochondria play a crucial role in regulating cellular energy metabolism [12]. Members of the COX family are included in the electron transport chain complex IV, modulating mitochondrial morphology and function, which is vital for cellular activities [22]. As shown in Fig. 5H, our work revealed that COX5B was overexpressed and predicted as an independent prognostic marker in LUAD. Mechanistically, we showed that COX5B regulated the ATP and ROS levels to promote the proliferation of LUAD cells. We also validated that berberine could target COX5B to inhibit the LUAD progression. This might provide a new strategy for LUAD clinical treatment.

Recently, growing studies have suggested that COX5B influences tumor development through the regulation of mitochondrial function. Heat shock protein family D member 1 (HSPD1) promoted the progression of non-small cell lung cancer (NSCLC) dependent on SLC6A8 and COX5B [23]. Three compounds (gallic acid, betulinic acid, and caffeic acid) had a significant inhibitory effect on lung squamous cell carcinoma via five biolabels (CPS1, CKM, CPT1B, COX5B, and COX4I1) [24]. It was observed that the expression levels of DARS2 and COX5B were significantly higher in the LUAD than in adjacent normal tissue and correlated with the LUAD patients’ prognosis. Knockdown DARS2 and COX5B inhibited tumor cell proliferation [25]. The consistent results were obtained in our own study. We also identified that COX5B was a reliable prognostic marker for LUAD patients via several prognostic models. Moreover, we have conducted further in-depth research, mainly in the following three aspects: (1) Several experiments together with bioinformatics analysis proposed that COX5B regulated the ATP and ROS levels to promote the proliferation of LUAD cells. (2) Through various computational algorithms, we also found that COX5B hampered immune infiltration in LUAD. (3) Our study is the first to demonstrate, both *in vitro* and *in vivo*, that berberine suppressed LUAD cell proliferation and tumor growth by targeting COX5B, offering a potential therapeutic target and strategy for clinical treatment.

Our study found that downregulation of COX5B caused impaired oxidative phosphorylation (OXPHOS). This discovery is closely related to the classical Warburg effect—a phenomenon where cancer cells preferentially rely on glycolysis [26]. We thought that COX5B deficiency might not merely be a consequence of OXPHOS inhibition but could act as a driver of metabolic reprogramming. On one hand, dysfunction in OXPHOS and the TCA cycle might lead to the accumulation of metabolites such as succinate, which inhibits PHD activity and stabilizes HIF-1α [27]. On the other hand, ROS also served as signaling molecules to activate HIF-1α [28]. HIF-1α is a key transcription factor that upregulates glycolysis-related genes, including GLUT1, HK2, LDHA, ultimately promoting the Warburg effect. Therefore, COX5B might function as a critical metabolic switch to influence the energy balance between OXPHOS and glycolysis in cancer cells. Future studies should directly measure the glycolytic rate (e.g., ECAR) upon COX5B knockdown and validate the activation status of HIF-1α.

As is well known, the tumor development is closely linked to the surrounding immune microenvironment [29,30]. Containing immune cells, inflammatory cells, blood vessels, and extracellular matrix, the immune microenvironment plays an important role in tumor progression and response to therapy [31,32]. Krupar et al. found that COX5B and GLUT1 were increased, while intratumoral CD8/CD4 was decreased [33]. Pearson and ssGSEA analyses showed that the expression of genes (NDUFB8, COX6C, NDUFA6, USMG5, and COX5B) had a significant negative correlation with the proportion of Tcm immune cells [34]. Consistently, we observed that COX5B was negatively related to the infiltration of Tcm and Tem cells. These results suggest an association between COX5B and suppressed immune infiltration. However, there is a lack of direct evidence that COX5B inhibits immune cell infiltration. This remains a key area for future research.

These days, reusing the existing medicine for cancer treatment has garnered significant interest due to the established safety, dosage, and toxicity profiles [35]. Stiripentol, an inhibitor of lactate dehydrogenase A, was used in epilepsy management to markedly decrease the lactate levels and lead to the reverse chemoresistance [36]. Serotonin (5-HT) is a neurotransmitter whose deficiency is linked to depression. Recent research indicated that 5-HT facilitated tumor progression through immunosuppressive mechanisms [37]. Here, we knocked down COX5B in LUAD cells. The results of electron microscopy revealed that the mitochondria were swelling and rupturing, and the loss of cristae was shown in the knockdown group, alongside decreased ATP levels and elevated ROS levels. Through compound library screening and molecular docking, we discovered that berberine can specifically associate with COX5B and decrease its protein expression. Berberine targets COX5B to inhibit the proliferation of LUAD cells *in vitro* and tumor growth *in vivo*. Consistently, other researchers have also demonstrated that berberine can inhibit oxidative phosphorylation in non-small cell lung cancer cells and tumor-associated fibroblasts [38]. Inhibitors of oxidative phosphorylation, such as Gboxin and berberine, have been shown to impede the growth of mouse tumor xenografts. Retrospective analyses of clinical tumor samples have indicated that berberine administration leads to a lower recurrence rate of NNMT1^low^/DNMT1^high^ colorectal adenomas [39].

While our study provides compelling evidence for the role of COX5B in LUAD, several limitations should be acknowledged. Firstly, the lack of validation of the berberine’s effect on tumor growth in several preclinical LUAD models (patient-derived tumor xenograft, organoids) was a major limitation. Secondly, the number of animals used in the experiment was only five per group. This should be increased in future studies to enhance the reliability of the data. Lastly, we only performed the bioinformatics analysis and IHC assays to suggest that COX5B was negatively related to immune infiltration in LUAD. The functional immune assays were absent. In the following study, it is essential to construct an orthotopic tumor model with COX5B-knockdown and control LUAD cells to analyze the differences in immune cell infiltration using flow cytometry or single-cell sequencing.

In conclusion, our findings reveal the promoting role of COX5B in LUAD by bioinformatics analysis, together with experimental validation. Moreover, our study also highlights the potential of targeting COX5B by berberine for the treatment of LUAD. Further research is needed to explore the precise mechanism and clinical application of COX5B-targeted therapies in LUAD.

## Supplementary Materials



## Data Availability

All data generated or analyzed during this study are included in this published article (as well as in the accompanying Supporting Information).

## Supplementary Materials

The supplementary material is available online at https://www.techscience.com/doi/10.32604/or.2025.069889/s1.
