# Supplementary material for: Integrative Multi-Omics Analysis and Experiments Validation Identify COX5B as a Novel Therapeutic Target for Lung Adenocarcinoma
Source: Oncol Res. 2025 Dec 30;34(1):22. doi: 10.32604/or.2025.069889 (PMC12774556; doi:10.32604/or.2025.069889)
Supplement: Supplementary file 1 [file OncolRes-34-69889-s001.docx]

**Figure S1:** The expression pattern and function of COX5B in pan-cancers. The TCGA database (A) and UALCAN database (B) were employed to discover the expression pattern of COX5B in pan-cancers. The relationship between COX5B and overall survival (C), progression-free interval (D), disease-specific survival (E), and immune cells infiltration (F) in pan-cancers was estimated from the TCGA database. * *p*<0.05, ** *p*<0.01, *** *p*<0.001. ACC: adrenocortical carcinoma, BLCA: bladder urothelial carcinoma, BRCA: breast invasive carcinoma, CESC: cervical squamous cell carcinoma and endocervical adenocarcinoma, CHOL: cholangiocarcinoma, COAD: colon adenocarcinoma, DLBC: lymphoid neoplasm diffuse large B-cell lymphoma, ESCA: esophageal carcinoma, GBM: glioblastoma multiforme, HNSC: head and neck squamous cell carcinoma, KICH: kidney chromophobe, KIRC: kidney renal clear cell carcinoma, KIRP: kidney renal papillary cell carcinoma, LAML: acute myeloid leukemia, LGG: brain lower grade glioma, LIHC: liver hepatocellular carcinoma, LUAD: lung adenocarcinoma, LUSC: lung squamous cell carcinoma, MESO: mesothelioma, OV: ovarian serous cystadenocarcinoma, PAAD: pancreatic adenocarcinoma, PCPG: pheochromocytoma and paraganglioma, PRAD: prostate adenocarcinoma, READ: rectum adenocarcinoma, SARC: sarcoma, SKCM: skin cutaneous melanoma, STAD: stomach adenocarcinoma, TGCT: testicular germ cell tumors, THCA: thyroid carcinoma, THYM: thymoma, UCEC: uterine corpus endometrial carcinoma, UCS: uterine carcinosarcoma, UVM: uveal melanoma.
